# Arenavirus as a potential etiological agent of odontogenic tumours in humans

**DOI:** 10.1186/s13046-020-1540-1

**Published:** 2020-02-10

**Authors:** Marco de Feo, Cristina De Leo, Umberto Romeo, Paola Muti, Giovanni Blandino, Silvia Di Agostino

**Affiliations:** 1 Department of Odontostomatological and Maxillo Facial Sciences, University of Rome La Sapienza, 00161 Rome, Italy; 2 Dental practioner and oral surgeon volunteer at Saint Mary’s Hospital, Lacor, Gulu, Uganda; 3 Clinical and Microbiological Chemical Analysis Laboratory, IRCCS Istituto Dermopatico dell’Immacolata-IDI, 00167 Rome, Italy; 4 Department of Biomedical, Surgical and Dental Sciences, University of Milan La Statale, 20122 Milan, Italy; 5 Oncogenomic and Epigenetic Unit, Department of Diagnostic Research and Technological Innovation, IRCCS Regina Elena National Cancer Institute, 00144 Rome, Italy

**Keywords:** Odontogenic tumors, Arenavirus, Lassa virus, Ameloblastoma, Ossifying fibromas, Fibrous bone tumors

## Abstract

Odontogenic tumors (OT) are considered rare events and their epidemiologic data are scarce and under-estimated in developing countries because there is no systematic collection of clinical features including histological analyses of the tissue samples. Furthermore, there is an underestimation of the disease relevance and affected people are often marginalized in spite of severe functional impairment of aero-digestive tract. Etiology of OT in humans is still unknown and it represents an important therapeutic and diagnostic challenge.

Lassa fever is an acute viral haemorrhagic illness caused by Lassa virus, a member of the arenavirus family of viruses. The disease is endemic in the rodent population in West-East Africa. Humans usually become infected with Lassa virus through exposure to the food or household items contaminated with urine or feces of infected rats. It is also reported person-to-person infections. About 80% of people infected by Lassa virus have no symptoms but the virus establishes a life-long persistent infection.

The present commentary significance is to start, for the first time ever, a systematic collection of clinical features and tissue sample collection at the St. Mary’s Hospital in Lacor (Gulu) North Uganda where the considered pathologies have an important frequency. The systematic collection will allow to corroborate the possible association between arenaviruses infection and pathogenesis of odontogenic tumors in humans.

## Background

Odontogenic tumors (OTs) are uncommon neoplastic lesions of the maxilla and mandible, which present difficult diagnosis and therapeutics. The majority of these lesions represent real neoplasms with a subgroup of them characterized by invasive behavior. Furthermore, studies have shown that the distribution and the frequency of this pathology presents geographic variations [1–3]. Currently, there is very little information from specific locations such as Uganda and retrospective published studies are very dated [3].

Among the OTs, it was documented in the sub-saharan local population cases regarding ossifying fibromas, ameloblastomas, fibrous dysplasia and odontogenic fibromixoma, which cause devastating facial deformations of children and young adults [3]; the surgical intervention leaves deformed faces condemning patients to the isolation. About 70% of these tumors originate in the head and neck region and the pathogenesis is unknown [1]. There is a broad scientific consensus reporting that these tumors are more frequent in developing countries as Uganda, Mozambique, Nigeria, Ghana, Benin, Zimbabwe, Tanzania and other sub-Saharan african countries rather than others, despite ethnic differences and genetic diversities [2].

**Fig. 1:**
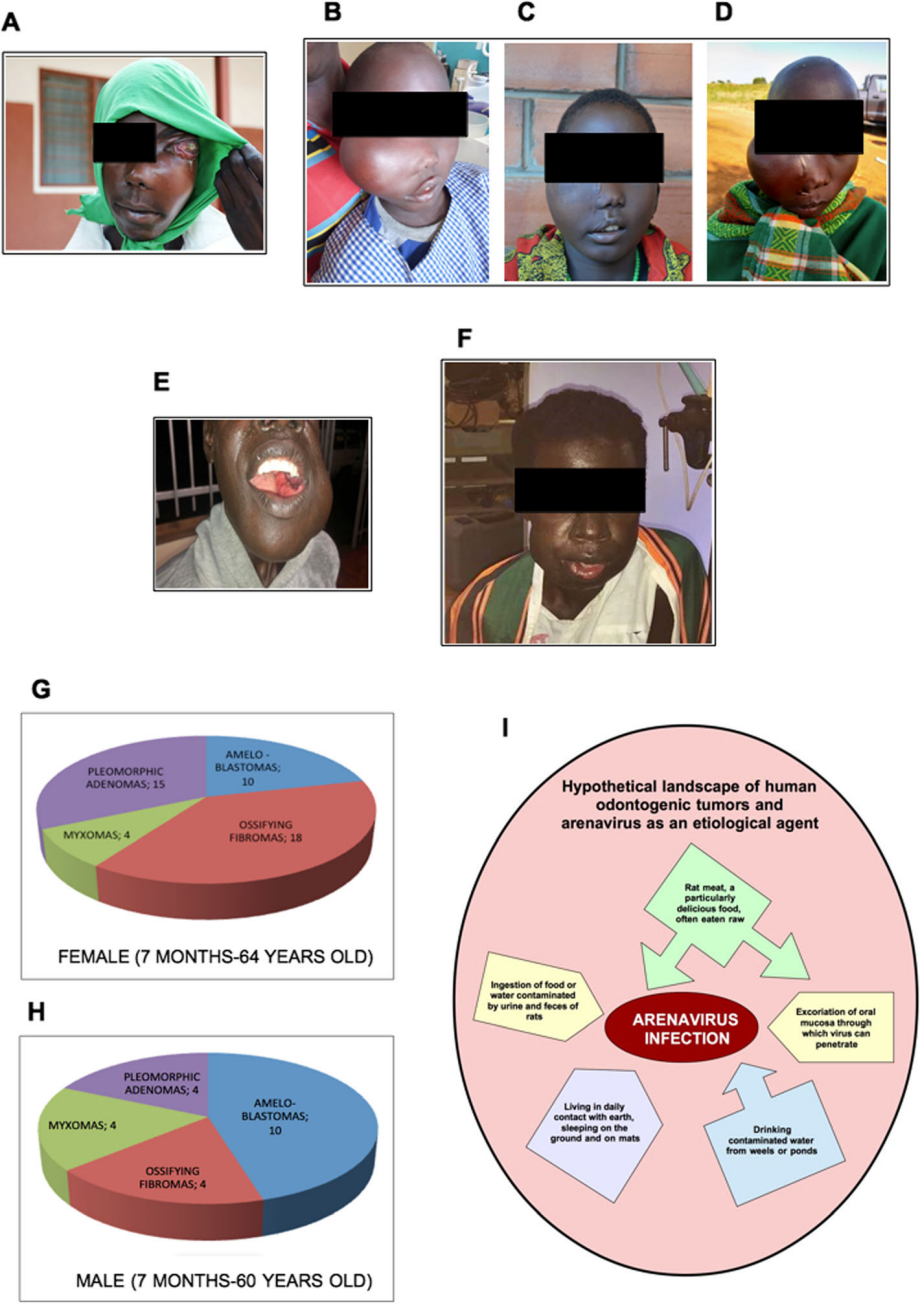
**a** Example of odontogenic fibromixoma in a young boy. **b** Six years old child from the ethnic group of Karimojong affected by ossyfing fibroma. The photo shows second surgical procedure’s results, due to a tumor recurrence. **c** The same six years old child presented in (**b**), after the third surgical procedure. **d** Again the same child, after six months from the third intervent. The tumor has recurred. **e** A woman affected by ameloblastoma before the third surgical procedure on the left side (First resection on 1998, the second on 2013, the third on September 2019). **f** The same patient captured only one month later the third surgical intervention, with a new tumor in the right side. Odontogenic tumors at Saint Mary’s hospital in 2018. **g** Number of odontogenic tumors in female patients. **h** Number of odontogenic tumors in male patients. **i** Scheme of arenavirus infection ways that may cause latent and persistent infection in the human’s oral cavity, causing odontogenic tumors

In the Uganda villages, people live in huts in contact with the earth, sleeping on the ground and on mats, often bitten by mice, drinking and cooking with water from wells or ponds where children and adults bathe, easily contaminated by urine and rat feces. The local populations consider rat meat as a particularly delicious food, main source of protein and iron, but rats are often eaten either raw or cooked on charcoal which doesn’t inactivate contaminating viruses [4]. Because of the hospitals are few and far from villages, patients reach them when facial deformities have severely progressed. We, as medical staff, often observed the presence of oral tumors in children living in these difficult conditions (Fig. 1a-f). Scientific reports on populations who consume rats, serpents and bats are scarce and only reported by newspapers and magazines (https://www.theeastafrican.co.ke/magazine/434746-247534-c5x5o9/index.html; https://www.newvision.co.ug/new_vision/news/1129419/rats-dinner-delicacy-taboo).

Lassa virus, the causative agent of Lassa fever, is a member of the family *Arenaviridae* and it continues to be the most common cause of hemorrhagic fever in West-Est Africa, infecting roughly 400,000–500,000 individuals per year and yielding a yearly death rate of 30,000–40,000 [5, 6]. The *Arenaviridae* family (Arenavirus) consists of two single stranded, ambisense RNAs. It causes chronic asymptomatic infections in rodents [5]. Humans are infected most likely through mucosal exposure to aerosols or by direct contact between infectious materials as rat meat and abrasion of the skin and oral mucosa. Arenavirus can move through both the blood and the lymphatic system, thus reaching the major and minor salivary glands, and more human-to-human infection and the passage of the virus from the mother to the placenta have also been documented [5, 6]. Arenaviruses are grouped in Old World as Lassa virus (LF) endemic in West Africa and in New World as Junin widespread in South America [5, 6].

Epidemiological data on arenavirus infections in Uganda are very scarce. Only a systematic serological survey in the Uganda Karamoja District supported the presence of Lassa virus infections in that district population [7].

## Main text

This study started at St. Mary’s Hospital in Lacor (Gulu) in the North of Uganda, a missionary hospital in the middle of the savanna which has been isolated for 27 years due to political unrest, lack of roads and poverty.

At St. Mary’s Hospital in 2018, the volunteer medical staff documented 47 cases of OTs in the female patients ranging from 7 months to 64 years old (Fig. 1g), and 22 cases of OTs in the male patients ranging from 7 months to 6 years old (Fig. 1h). Furthermore, we also observed a high number of adenomas in the major salivary glands which unfortunately were not systematically reported during the year 2018.

Recently it has been documented a large intraoral mass from the buccal gingiva in a captive bred red tail boa (*Boa constrictor constrictor*), diagnosed as odontogenic fibromyxoma very similar to those occurring in humans [8]. An arenavirus-like virus was detected in the neoplastic tissue and in the cancer recurrence 2 years later by using reverse transcription polymerase chain reaction (RT-PCR) [8]. This paper captured our attention as the finding was very striking to support the hypothesis of a possible role of arenaviruses in odontogenic fibromyxoma oncogenesis.

Several viruses, named oncovirus, may cause the developing of a cancer inducing alteration of gene expression, gene mutation or by suppressing the immune system causing long-term inflammation [9]. For example few types of Human papillomaviruses (HPVs) are the etiological agents of cervical cancer, Epstein-Barr virus (EBV) is a type of herpes virus and its life-long infection increases the risk of getting nasopharyngeal cancer and liver cancer [9].

A hallmark characteristic of arenavirus infection is its ability to establish life-long persistent infection avoiding to enter in a “latent phase” without adverse consequences such as retroviruses like HIV-1 or herpes viruses [6, 9]. In humans, LF is usually characterized by general flu-like symptoms as fever, malaise and headache, but in severe cases it can develop hemorrhaging and/or neurologic involvement that may be fatal [6, 9]. Viral evasion from the immune system is critical for productive replication and dissemination in the host. Arenavirus releases the evasion through the development of exhausted T cells (T^EX^) [6]. T^EX^ are defined by reduced effector function, sustained upregulation of multiple inhibitory receptors as PD-1, an altered transcriptional program and perturbations of normal memory development and homeostasis [6, 9]. Exhaustion was originally identified in CD8+ T cells (T^EX^) during lymphocytic choriomeningitis virus (LCMV, belonging to mammarenavirus) infection and subsequently in humans with HIV, hepatitis C virus (HCV), hepatitis B virus (HBV), and cancer [6, 9]. This biological event is characteristic of immune responses to chronic viral infections and cancer.

Thus, when HPV and EBV infections have been inferred in patients with gnathic and peripheral ameloblastomas the lack of evidence of their infection leads to the exclusion of their etiopathogenetic role in ameloblastoma [10]. This data reinforces the idea of testing the presence of arenavirus in tissues deriving from OT patients (Fig. 1i).

## Conclusions

To date, no research has shown a correlation between persistent arenavirus infection and human neoplasm development. Several oncoviruses, inducing long-term infections, have been linked to an increased cancer risk. Since arenavirus causes latent/persistent inflammation, we suggest new systematic collection of data in countries at high prevalence of arenavirus infection to test the hypothesis that these viruses are associated with OT development in humans.

Only for Junin in South America exists the vaccine Candid#1 from 1990. Unfortunately, LF affected african countries where the socio-economic problems, the lack of systematic clinical data registration and the poverty make difficult the development of a vaccine and to attract the commitment of pharmaceutical companies willing to invest in such an enterprise.

To give consistency to the hypothesis that arenaviruses may be the etiological agents of odontogenic fibrous bone tumors, it is necessary to systematically collect tissue samples and clinical informations from patients through the collaboration of local medical staff in order to test the virus infection and to allow the production of antiviral treatment for susceptible individuals (Fig. 1i).

## Data Availability

Not applicable.

## Abbreviations

EBV: Epstein-Barr virus
HBV: Hepatitis B virus
HCV: Hepatitis C virus
HPV: Human papillomavirus
LCMV: Lymphocytic choriomeningitis virus
LF: Lassa virus
OT: Odontogenic tumor
